# Mass Spectrometric Analyses of Organophosphate Insecticide Oxon Protein Adducts

**DOI:** 10.1289/ehp.0900824

**Published:** 2009-07-29

**Authors:** Charles M. Thompson, John M. Prins, Kathleen M. George

**Affiliations:** Center for Structural and Functional Neuroscience, Department of Biomedical and Pharmaceutical Sciences, University of Montana, Missoula, Montana, USA

**Keywords:** acetylcholinesterase, adduct, butyrylcholinesterase, insecticide, mass spectrometry, organophosphate, organophosphorus, peptide, protein

## Abstract

**Objective:**

Organophosphate (OP) insecticides continue to be used to control insect pests. Acute and chronic exposures to OP insecticides have been documented to cause adverse health effects, but few OP-adducted proteins have been correlated with these illnesses at the molecular level. Our aim was to review the literature covering the current state of the art in mass spectrometry (MS) used to identify OP protein biomarkers.

**Data sources and extraction:**

We identified general and specific research reports related to OP insecticides, OP toxicity, OP structure, and protein MS by searching PubMed and Chemical Abstracts for articles published before December 2008.

**Data synthesis:**

A number of OP-based insecticides share common structural elements that result in predictable OP–protein adducts. The resultant OP–protein adducts show an increase in molecular mass that can be identified by MS and correlated with the OP agent. Customized OP-containing probes have also been used to tag and identify protein targets that can be identified by MS.

**Conclusions:**

MS is a useful and emerging tool for the identification of proteins that are modified by activated organophosphate insecticides. MS can characterize the structure of the OP adduct and also the specific amino acid residue that forms the key bond with the OP. Each protein that is modified in a unique way by an OP represents a unique molecular biomarker that with further research can lead to new correlations with exposure.

The term “organophosphate” (OP) is often used in the scientific and lay press to describe a large chemical class of insecticides and chemical warfare agents. OP insecticides, which include malathion and chlorpyrifos, among others, are among the most widely used agrochemicals for the control of insect pests in the world. Approximately 427 tons of OP insecticides were used for vector control in 2003–2005 (Zaim and Jambulingam 2007), and > 36.5 million tons were used in agriculture in the United States in 2001 [Barr et al. 2005; Centers for Disease Control and Prevention 2005; U.S. Environmental Protection Agency (EPA) 2006]. This level of use inevitably leads to increased human exposure and toxicity incidences both in the United States and worldwide (Claudio et al. 2000; Goldman and Koduru 2000; Menzies and MacConnell 1998). A total of 136,881 OP exposure incidents were reported to poison control centers in the United States during 1995–2004 (Sudakin and Power 2007), and in 2007 the American Association of Poison Control Centers received 96,307 calls associated with OP exposure (Bronstein et al. 2008).

The principle trigger of neurotoxicity after exposure to OPs is presumed to result from the inhibition of acetylcholinesterase (AChE) (Ballantyne and Marrs 1992; Broomfield et al. 1995; Eto 1974; Fest and Schmidt 1973; Fukuto 1990). When AChE is inhibited by an OP, it is unable to hydrolyze the neurotransmitter acetylcholine (ACh), which reaches toxic concentrations in the neural synapse, causing hyperstimulation of cholinergic receptors, resulting in tremors, lacrimation, and bradyarrhythmia and, if untreated, may become lethal (Costa 2006; Sultatos 1994). A number of noncholinergic illnesses have also been linked to OPs, including ataxia, delayed neuropathy, intermediate syndrome, pulmonary toxicity, genotoxicity, Parkinson disease, and vision loss (Arima et al. 2003; Dahlgren et al. 2004; Jayawardane et al. 2008; Niven and Roop 2004; Yanagisawa et al. 2006; Zwiener and Ginsburg 1988). Widely reported but unsubstantiated health problems associated with OP exposure include flu-like symptoms, nausea, weakness, and dizziness (Gordon 1993; Rowsey and Gordon 1999; Solomon et al. 2007; Stephens et al. 1995). Low-dose OP exposure causes minimal inhibition of AChE and no obvious cholinergic symptoms yet has been linked to memory loss, sleep disorder, depression, learning and language impairment, and decreased motor skills in humans (Johal et al. 2007; Lockridge and Masson 2000; Slotkin et al. 2008; Timofeeva et al. 2008). Despite these numerous reports of OP-associated illnesses, few if any have been correlated with a specific protein, pathway, or cellular event that has been modified, disrupted, or regulated by an OP.

Recent studies have associated adverse neurologic and growth outcomes in children exposed to certain OPs *in utero* (Jacobson and Jacobson 2006; Whyatt et al. 2004, 2005). A neuropsychological assessment of children exposed to OPs showed deficits in inhibitory motor control (Kofman et al. 2006). Two associated studies correlated developmental exposure with abnormal reflexes (Young et al. 2005) and mental developmental problems (Eskenazi et al. 2007). OPs are thought to cause developmental neurotoxicity and long-term cognitive and behavior effects through cholinergic mechanisms, interference with nonenzymatic functions of AChE, and effects on cell signaling pathways involved in neural cell differentiation (Slotkin 2004).

Many claims of illness due to OP-based exposure have not been correlated with dose largely because of inadequate methods of analysis and the transient nature of OP compounds. The environmental lifetime of OP insecticides is typically considered to be rapid (days), but in indoor applications certain OPs can survive intact from months to years (Fenske et al. 2000). Still, the rapid breakdown of OPs *in vivo* or in the environment makes their direct measurement challenging, and rough estimates or indirect measures of exposure are conducted by identification of OP metabolites or degradation products. For example, gas chromatography (GC) coupled with mass spectrometry (MS) has been used to analyze the primary metabolites of OP compounds, typically the hydrolyzed acids of dialkylphosphates [RO_2_P(O)OH] in urine (Barr et al. 2005).

For decades, exposure to OP insecticides has been determined using a blood cholinesterase test, a colorimetric assay in which a reduction in basal cholinesterase activity in whole blood or serum is used to indicate OP exposure (Ellman et al. 1961). Plasma butyrylcholinesterase (BChE) assays are used for the early, acute effects of OP exposure because red blood cell AChE assays are less sensitive (Wilson et al. 1996). By design, the blood cholinesterase test measures only the “active” form of the enzyme, but it is the inactive or OP-adducted form of the enzyme that is the more quantifiable biomarker.

To address this, investigators have begun to employ computational, high-throughput, bioinformatic, and proteomic methods to identify and quantify molecular biomarkers to a number of environmental- and exposure- based diseases. For this review we identified general and specific research reports related to OP insecticides, OP toxicity, OP structure, and protein MS by searching PubMed (National Center for Biotechnology Information 2009) and Chemical Abstracts (Chemical Abstract Service, Columbus, OH) for articles published before December 2008. Here, we summarize the use of protein MS as a valuable tool to identify OP-adducted proteins and as a potentially promising approach to deconvolute a number of OP-triggered illnesses. We also discuss MS analysis of proteins adducted by chemical nerve agents and the use of customized probes for identification of OP-modified proteins. To focus our discussion, we do not include genomic studies of protein regulation changes resulting from OP exposure or the MS of OP compounds.

## Structure and Reactivity of OP Compounds

The overall class of organophosphorus (V) compounds includes a number of useful structure types, among which are the OPs, which are functional group derivatives of phosphoric acid and include ester (phosphate), amide (phosphoramidate), thiolester (phosphorothiolate), difunctional and trifunctional forms, and combinations of these heteroatomic linkages. Unlike the structurally similar carboxylic acids, however, OP compounds can vary as phosphoryl (P=O) or as thiophosphoryl (P=S). These subtle atomic differences lead to a wide array of structural types that vary dramatically in their chemical, physical, and biochemical properties. Importantly, the “ate” ending associated with heteroatom-attached groups designates the structure as a phosphoric acid functional group derivative. This description also includes structures containing one phosphorus–carbon (P–C) group, termed a phosphonate, and two P–C groups, termed a phosphinate. When three P–C bonds occur, the structure is no longer a phosphoric acid derivative and there is no “ate” ending (e.g., Ph_3_P=O is triphenylphosphine oxide). Because most insecticides contain three phosphorus-heteroatom groups, the term “organophosphate” is both useful and chemically accurate for the purposes of this review. However, “organophosphorus insecticide” is a useful description and superior when searching certain databases.

**Figure 1:**
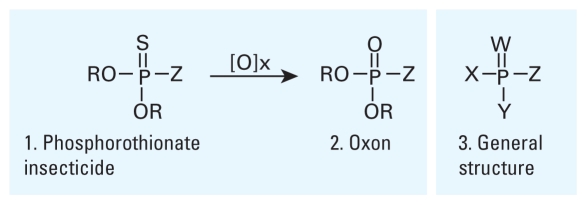
Structure of OP insecticides and select OP agents. Abbreviations: Et, ethyl; Me, methyl; Ph, phenyl. For malathion, R = Me, Z = SCH(CO_2_Et)CH_2_CO_2_Et; parathion, R = Et, Z = O–Ph–*p*–NO_2_; chlorpyrifos, R = Et, Z = *o*-2,3,5-trichloropyridinol; azinphos, R = Et, Z = SCH_2_–3-benzenetriazole-4-one; diazinon, R = Et, Z = O[6-Me, 4-iPr-pyrimidinol]. Parathion, chlorpyrifos, azinphos, and diazinon are also available as dimethyl esters.

Malathion, parathion, chlorpyrifos, azinphos, and diazinon are representative OP insecticides (Figure 1, structure 1). These OP insecticides are structurally similar and share diester and thionate (P=S) groups. For example, parathion, chlorpyrifos, and diazinon are diethyl phosphorothionates that vary only in the leaving group (Z; see Figure 1). Malathion and azinphos have a thiol as the leaving group. When both P=S and P–SR groups are present, the structure is a thiolothionate, or the more general term, dithioate. Although an extensive examination of OP insecticide structure is beyond the scope of this review, it is important to note that when leaving group differences are ignored, most OP insecticides fall into just two general classes: dimethoxy OP (DMOP) and diethoxy OP (DEOP). A smaller fraction of OP insecticides do not share these structural traits; acephate, for example, is a phosphoramidate with an oxon (P=O) bond and a phosphoramide (P–N) bond.

**Figure 2:**
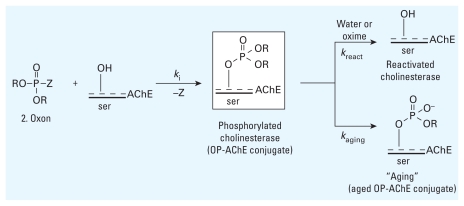
Formation of OP oxon and reactions with AChE. Abbreviations: *k*_aging_, aging (nonreactivation) rate constant; *k_i_*, inhibition rate constant; *k*_react_, reactivation rate constant.

For OP insecticides to impart their action, they first require a change in structure from a thionate to an oxon (structures 1 and 2, respectively, in Figure 1). The oxon is markedly more reactive and likely the primary chemical species responsible for most interactions with biomolecules as well as a key intermediate in route to hydrolytic metabolites. After an OP insecticide is converted to the oxon, it is known to react quickly with certain proteins to form OP adducts (Figure 2). A governing property of most OPs, therefore, is that they covalently modify proteins to form OP-adducted proteins in which protein function can be compromised concomitant with an increase in the protein’s molecular weight due to the addition of the OP. Covalent modification or organophosphorylation of a protein is a distinctive process because most organic and inorganic compounds transiently bind or form complexes with proteins but less frequently form a covalent bond with the protein.

Phosphorylation by insecticides is best exemplified by the inhibition of the primary target of OP insecticides, AChE (Figure 2). OP inhibition occurs at an essential serine (ser) residue synchronous with the ejection of a leaving group (Z) to yield a stable covalent bond, forming an OP–AChE adduct. OP-inhibited AChE can then reactivate (*k*_react_) via cleavage of the phosphoserine bond (Ashani et al. 1990, 1995, 1998; Langenberg et al. 1988; Lanks and Seleznick 1981; Wilson and Henderson 1992; Wilson et al. 1992) or undergo “aging” (Harel et al. 1991), a slow, irreversible process (*k*_aging_ << *k_i_*) that results in an OP–AChE adduct that contains a phosphate oxyanion (Doorn et al. 2000; Lotti et al. 1984).

The overall mechanism of AChE inhibition, reactivation, and aging by OP oxons is highly conserved because most OP insecticide oxons bear either dimethoxy or diethoxy groups. Therefore, loss of the leaving group Z affords two predominant forms of OP–AChE adducts: the DMOP (R=Me) and DEOP (R=Et). DEOP-adducted AChE is expected to be more stable and slower to reactivate and should undergo aging (correlated with the increased size of the R group). The dimethoxy analog should reactivate more rapidly and undergo less aging, although methyl esters undergo hydrolysis more rapidly than do ethyl esters. OP insecticide oxons, therefore, adduct AChE to form DMOP–AChE and DEOP–AChE structures that lead to two aged forms, monomethoxy phosphoryl (Y=OMe) and monoethoxy phosphoryl (Y=OEt) (Figure 2), respectively. These convergent mechanisms furnish investigators with some preliminary molecular targets for defining the OP proteome (the complement of proteins found in cells, tissues, or organisms that react with OP compounds) because the overall mechanism of organophosphorylation is most likely conserved for most other proteins (e.g., receptors, enzymes). In a like manner, OP oxons of any structure can modify AChE or any protein with a reactive residue if a suitable leaving group Z is present. In the case of the general OP structure (Figure 1, structure 3), loss of the Z group would generate a protein-bearing XYP(O) group attached to a serine.

A key difference is that biochemical phosphorylation and dephosphorylation, which are mediated by kinases and phosphatases, result in the addition and loss of an inorganic PO_4_^2−^ group. Although this is clearly different from the organophosphorylation process, it is entirely possible that OP insecticide oxons react with the same proteins that are substrates of kinases/phosphatases. Posttranslational phosphorylation is relatively well understood and is now a routine part of proteomics analysis (Godovac-Zimmermann et al. 1999; Larsen et al. 2001).

## The Search for OP Biomarkers: Possible Pathways

**Figure 3:**
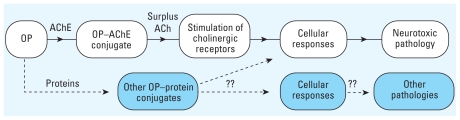
Common sequence of cholinergic events and alternate sequence after OP exposure. The white pathway (top) shows the stepwise phosphorylation of AChE (OP–AChE adduct), cholinergic hyperstimulation, and resultant cellular responses. The blue pathway (bottom) shows possible connections between OP–protein adducts other than AChE and the resulting cellular/toxic responses.

Toxic effects due to OP exposure likely begin with covalent modification (adduction) of a protein and thereby constitute an early biomarker of exposure. For example, the well-documented OP neurotoxic pathologies are believed to result either directly or indirectly from stepwise phosphorylation of AChE (OP–AChE adduct), cholinergic hyperstimulation, and resultant cellular responses (Figure 3, top pathway). However, the connection between OP–protein adducts other than AChE and the resulting cellular/toxic responses have not been established (Figure 3, bottom pathway). A clearer understanding of how alternate cellular pathways are modulated, altered, or blocked in response to OP exposure is needed so that more accurate, pathway-oriented biomarkers can be identified.

More recently, investigators have begun to investigate covalent OP adduction of non-AChE protein targets as a possible causative step in other toxic responses. The involvement of OPs at individual ACh receptors (nAChR, mAChR) and noncholinergic protein targets has been reported with increasing frequency (Albuquerque et al. 1988; Bomser and Casida 2001; Corrigan et al. 1994; Ehrich et al. 1994; Eldefrawi and Eldefrawi 1983; Huff et al. 1994; Katz and Marquis 1992; Li et al. 2000a, 2000b; Pala et al. 1991; Quistad and Casida 2000; Quistad et al. 2000, 2001; Richards et al. 1999; Schuh et al. 2002; van den Beukel et al. 1998; Ward et al. 1993; Ward and Mundy 1996). However, only a few investigations have identified OP-altered protein targets, despite the clear evidence suggesting involvement in toxic mechanisms. In a biomarker search, investigators can point to pathways that individually or collectively may correlate with various mechanisms of toxicity (e.g., Figure 3). Overall, OP compounds react with AChE to form an OP–AChE adduct (Figure 3, top pathway) and also with other yet uncharacterized and unidentified proteins to form OP–protein adducts that trigger new cellular outcomes (Figure 3, bottom pathway) and/or produce OP-adducted biomolecules that directly modulate, regulate, or shut down biochemical pathways (Bomser and Casida 2000, 2001; Bomser et al. 2002). Clearly, integrative proteomic and genomic approaches are needed to characterize these OP–protein biomarkers and validate them with any downstream effects. Such information would be invaluable for therapeutic intervention; body burden and tolerance; identification of genetic, age, race, and sex susceptibility; exposure prevention; and the overall assessment of OP safety.

## Protein MS

Proteomics and protein MS are powerful diagnostic methods to analyze and identify proteins and their modifications based on the molecular weight of peptide fragments resulting from enzymatic digestion. These methods can be conducted with a variety of mass analysis instruments, of which matrix-assisted laser desorption ionization (MALDI) and quadrupole-time of flight (QTOF) are among the most common in OP–protein studies. MALDI offers high-throughput, medium-resolution mass analysis (fingerprinting) of peptides and can also mass identify proteins after formulation with a matrix. MALDI is relatively easy to use and requires no chromatographic separation of the peptide digest, although separation of peptides can greatly assist identification. Most QTOF instruments use electrospray ionization (ESI) and allow for direct injection or use of HPLC (high-performance liquid chromatography) for separation of peptide analytes before MS. QTOF instruments are high-resolution instruments that can identify peptides using sequence information to provide greater statistical validation of both peptide and protein. Ion-trap and triple-quadrupole mass spectrometers are other instruments used in proteomics and protein MS experiments. Although MALDI and QTOF instruments use databases to identify peptides and the protein, the sequence information and mass accuracy provided by the QTOF ensure a more accurate peptide match and protein identification. An additional consideration in choice of mass spectrometer is the mass-to-charge ratio (*m*/*z*), which plays a role in the size of the peptide that can be analyzed accurately. Standard MALDI experiments detect single-charged peptides, meaning the instrumental mass observed correlates with the peptide mass. QTOF electrospray instruments (and other ion-based MS techniques) can detect singly, doubly, and triply charged peptide ions (e.g., a peptide weighing 4,200 *m*/*z* affords 2,100 *m*/*z* for a doubly charged ion and 1,400 *m*/*z* for a triply charged ion), which can be an advantage in the identification and sequencing of large-molecular-weight peptides.

**Figure 4:**
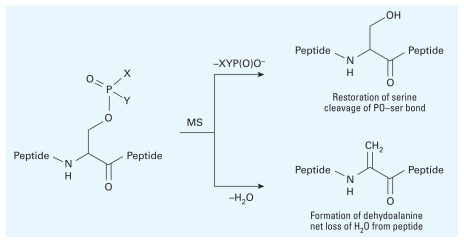
Dephosphorylation of peptides.

Both instruments are useful to analyze OP-modified protein adducts, but certain ionization methods (or cone voltages) used by electrospray MS instruments may cause cleavage of the somewhat labile phosphoester bonds formed with serine, threonine, or tyrosines (Figure 4). The loss of a (posttranslational) phosphate group from a peptide during electrospray MS analysis (X=Y=O^−^) can restore the serine residue or cause an elimination reaction that results in a dehydroalanine residue via the net loss of a water molecule (*m*/*z* 18) from the peptide (Figure 4). In addition to ionization conditions, the matrix used in MALDI may play a role in the stability of phosphoester linkage and its mass analysis. Several key studies and useful reviews have been published on MS-based dephosphorylation (reviewed by Sickmann and Meyer 2001) and will be helpful to those seeking to investigate OP–protein adducts (Adamczyk et al. 2001; Bennett et al. 2002; Jiang and Wang 2004; Qian et al. 2003; Thaler et al. 2003; Tseng et al. 2005). The use of barium hydroxide [Ba(OH)_2_] to induce formation of the dehydroalanine residue as a method to identify phosphorylation (Noort et al. 2006) is discussed below (see “MS Analysis of OP-Modified Proteins in Addition to Cholinesterases”).

## MS of Cholinesterases and Their Active Site-Containing Peptides

Historically, AChE and BChE are the principal targets covalently modified by OP compounds. Specifically, covalent modification of the essential serine residue (Figure 2) on AChE is of prime importance in MS analyses because this step initiates neurotoxic events. OP attachment to the serine residue represents the predominant if not definitive chemical pathway for analysis. Therefore, the molecular events that define esterase OP inhibition (including reactivation and aging; Figure 2) are likely to be similar to serine hydrolases, serine proteases, and other related proteins. Still, the assumption that serine is the only location for modification by OPs may be invalid, and steps are needed to develop methodology capable of identifying other OP-residue modifications.

First, it is important to show how OP–ChE adducts are identified by MS. In a typical experiment, a cholinesterase is inhibited by an OP; the protein–ChE adduct is isolated; the protein is digested into peptide fragments (trypsin, chymotrypsin, etc.); the peptides are analyzed by MS; and the modified OP–peptide is identified using a database match. A high degree of conservation in the primary sequences of AChE and BChE has enabled rapid identification of OP-modified peptides from various species. Tied to this advantage is the obvious site of OP modification: the catalytically active site serine. Unlike most investigations that require near complete N- to C-termini analysis to find one or more adducts, investigators have assumed that OP modification modifies only the active site serine hydroxyl. These two structural assumptions regarding cholinesterase reactivity toward OPs allowed investigators to narrow their mass analysis (ion selection) to a handful of peptide fragments, although multiple OP adducts are possible. Key to the OP–ChE protein adduct detection by MS, therefore, is the analysis and identification of the corresponding OP-modified active-site peptide.

**Figure 5:**
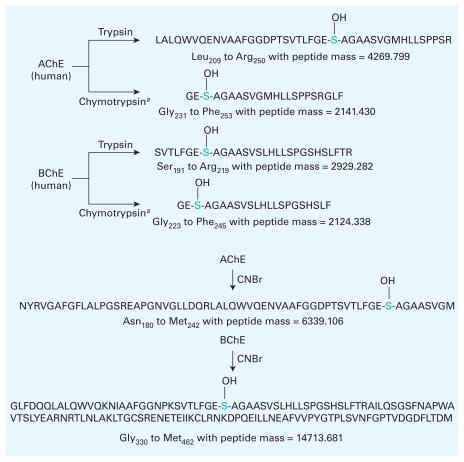
Active site-containing peptides resulting from trypsin, chymotrypsin, and CNBr digests of human AChE and BChE. Cleavages were simulated and masses calculated using ExPASy PeptideCutter (Swiss Institute of Bioinformatics 2007). The active site serine is indicated in blue. *^a^*Chymotrypsin is high specificity.

Although there are many methods for protein digestion, trypsin [cleaves at arginine (R) or lysine (K) at the C-terminus], pepsin [cleaves at phenylalanine (F), tryptophan (W), or tyrosine (Y) at the N-terminus], and chymotrypsin (cleaves at F, W, or Y at the C-terminus) are the most widely used peptidases. Cyanogen bromide [CNBr; cleaves at methionine (M)] has been used for chemical digestion before or after protease action. Key differences in the primary sequences of AChE and BChE result in active site-containing peptide fragments (Figure 5) of different sizes after trypsin and chymotrypsin digests. Different sources of cholinesterase (horse, rat, mouse, fly, eel, etc.) will produce different peptide molecular weights because the sequence is not 100% conserved.

Likewise, CNBr chemical digestion produces peptides containing the active site; however, the methionine groups are spaced relatively far apart in the sequence. This results in larger peptides (Figure 5) that can be more difficult to analyze and identify. However, the combination of chemical and peptidase digestions can be a powerful dual method for peptide analysis. In sum, understanding the predicted peptide fragments resulting from different digestion procedures allows researchers to plan their identification strategies.

There have been few attempts to map the entire AChE or BChE primary sequence using MS. Spaulding et al. (2006) sequenced a purified source of recombinant mouse AChE (rMoAChE) by ESI QTOF MS and found that the highest protein coverage (63%) and active-site peptide signal were achieved when the AChE:chymotrypsin ratio was 5:1. Some excellent early uses of MS to understand ChE structure were conducted by Rosenberry and colleagues, who deciphered the glycoinositol phospholipid anchor and protein C-terminus of BChE (Haas et al. 1996; Haas and Rosenberry 1995; Roberts et al. 1988).

One of the more recent benefits of MS is the ability to directly measure the entire protein mass and, in turn, the possibility of directly measuring OP-adducted proteins by the corresponding increase in the native mass. Mechanistic studies such as these are facilitated when cholinesterase can be expressed in good quantity and pure form, which reduces interferences from more abundant proteins typically found in biological matrices. One of the earliest such studies directly measured human AChE (hAChE) mass at approximately 64,700 Da (calculated at 64,695 Da) and OP-adduct masses increased as a function of inhibitor structure (Barak et al. 1997).

## Identification of OP-Modified Cholinesterases by MS

**Figure 6:**
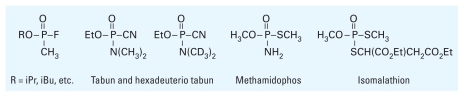
Structures of OP chemical agents and insecticide.

MS was used in early studies of mass shifts of AChE after OP exposure, including studies of chemical agents (Barak et al. 1997), insecticide and chemical agents (Elhanany et al. 2001), and insecticide impurities (Doorn et al. 2000). In these studies, the masses of whole protein and/or that of the active-site peptide were compared with the mass when bound to OP (less its leaving group). The influence of alkyl group size on aging (loss of an alkyl phosphoester group after inhibition) (Figure 6) was investigated using ESI‐MS (Barak et al. 1997). Recombinant hAChE (64,700 Da) was inhibited by methylphosphonate analogs, and the corresponding adducts CH_3_(RO)P(O)–hAChE, where R = isopropyl (iPr), isobutyl (iBu), 1,2-dimethylpropyl, and 1,2,2-trimethylpropyl showed measured mass increases of approximately 120, 140, 152, and 160 Da, representing the molecular weight of the added phosphonate group. Over time, each of the methylphosphonate adducts lost its alkoxy group to aging and converged to a mass of 64,780 Da, a value that structurally correlates with formation of a P(O)(CH_3_)OH adduct. Aging and reactivation are intimately tied mechanisms for most OP inhibitors of AChE and they have direct application to OP insecticide inhibitors bearing alkoxy groups.

Certain chemical agents and insecticides share the common structural feature of phosphoramide (P–N) group that can undergo aging. MALDI-TOF was used to identify the active-site peptide of AChE after trypsin digestion (Elhanany et al. 2001) by the phosphoramides tabun (chemical agent) and methamidophos (insecticide) (Figure 6). In an experiment designed to exploit the resolving power of MS, a hexadeuterio analog of tabun was used to form OP–AChE adducts; after trypsin digestion, the active-site peptide adduct showed an increase of 6 mass units versus unlabeled tabun (Figure 6). Again, the peptide adducts were of identical mass. The mechanism of AChE inhibition by methamidophos, previously shown to lose a thiomethyl (Thompson and Fukuto 1982), was validated by observation of the corresponding (MeO)(NH_2_)P(O)–peptide adduct and then formation of the free active-site peptide after reactivation (Elhanany et al. 2001). Isomalathion, an impurity in the insecticide malathion (Figure 6), contains two stereocenters that cause the mechanism of inhibition to occur via two pathways involving the loss of different leaving groups (Berkman et al. 1993a, 1993b, 1993c). A series of investigations by MALDI analysis of the active-site peptide determined that a single stereoisomer of isomalathion produces aged AChE, that is, loss of both thioalkyl groups (Doorn et al. 2000, 2001a, 2001b).

**Figure 7:**
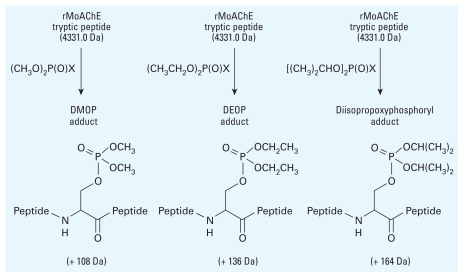
Formation of dialkoxyphosphate adducts of rMoAChE.

MALDI was used to study the covalent adduction of rMoAChE by a series of dialkoxy phosphates that varied as dimethoxy, diethoxy, and diisopropoxy (Jennings et al. 2003). The tryptic active site-containing peptide for rMoAChE was identified at *m*/*z* 4331.0; inhibition by diethoxy paraoxon showed an increase in this native peptide by 136 *m*/*z*. Likewise, inhibition by dimethoxy and diisopropoxy reactive OP compounds yielded the corresponding adducts (Figure 7). The MALDI experiments were correlated with kinetic analysis in the identification of the corresponding aged adducts for each alkoxy form. The ratio of inhibited, aged, and uninhibited forms of dimethyl phosphorylated AChE was delineated by their MS molecular weight signatures with tissue from a single mouse treated with sublethal doses of metrifonate (a DMOP inhibitor).

**Figure 8:**
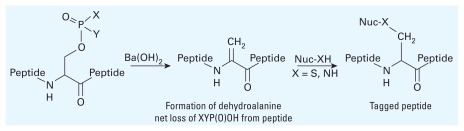
Ba(OH)_2_-induced dephosphorylation to tag proteins. Adapted from Noort et al. (2006). Nuc, nucleophile.

Chymotrypsin was used to digest rMoAChE into the smaller active-site containing peptide sequence GESAGAASVGMHIL (1298.62 Da) to more easily identify diethoxy and dimethoxy peptide adducts (Spaulding et al. 2006). A general method for detecting human OP–BChE adducts, reported by Noort et al. (2006), exploits the dehydroalanine- forming elimination mechanism from phosphoryl serines to tag proteins. BChE (from human plasma samples) that had been inhibited by OPs was digested with pepsin, converted to the dehydroalanine residue using Ba(OH)_2_, and reacted with amino or thiol nucleophiles to afford tagged peptides (Figure 8). The best results were achieved using 2-(3-aminopropylamino)ethanol to tag phosphylated BChE. The converted samples were then analyzed using liquid chromatography and tandem MS to determine the location of BChE modification by OPs. Because unmodified BChE does not form dehydroalanine, this method could be used to screen relatively large numbers of samples to detect OP exposure. The initial digest can then be analyzed more specifically to determine the identity of OP inhibitor.

## MS Analysis of OP-Modified Proteins in Addition to Cholinesterases

Cholinesterases are not the only targets of reactive OP compounds, and other molecular-level protein biomarkers have been identified with and without the aid of MS. Murray et al. (2005) found a number of adducted proteins in rat brain homogenates that were exposed to a variety of OPs (azamethiphos, chlorfenvinphos, diazinon, malathion, pirimiphosmethyl, and chlorpyrifos) at concentrations producing < 30% inhibition of brain AChE. Tritiated diisopropyl fluorophosphate was used to label protein targets not adducted by the test OP. Although the authors were not able to positively identify the protein targets, each of the six OPs tested adducted a different collection of proteins, suggesting that different OPs may produce their own specific form of toxicity (Murray et al. 2005).

**Figure 9:**
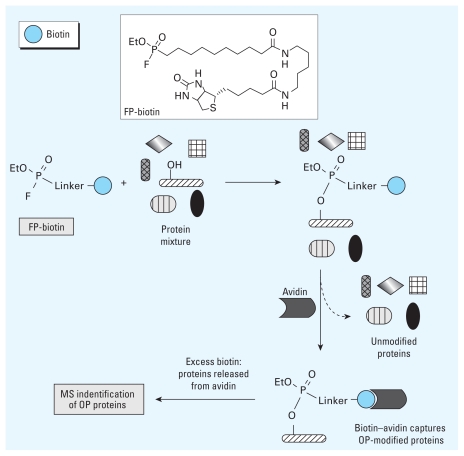
Structure of FP-biotin (a fluorophosphonate tethered to a biotin via a long linker) and the measurement of FP-biotin by MS to identify protein targets of OPs. An avidin affinity column captures the OP-biotin probe allowing unmodified proteins to pass through. OP-modified proteins bearing the biotin tag are released from the avidin column by treatment with excess biotin.

Hundreds of serine hydrolases are expressed in the human proteome, and many are potential targets for OP adduction. Synthesis of customized OP probes [fluorophosphonates (FPs)] that attach an OP to biotin (FP-biotin; Figure 9) or fluorescent molecules such as rhodamine permit “fishing” of cell lysates to access or identify non-AChE targets that were not previously identified (Casida and Quistad 2005). Examples of these enzymes include neuropathy target esterase, carboxylesterase, and platelet-activating factor acetylhydrolase as potential targets, as well as many other serine hydrolases. Nomura et al. (2006) identified serine hydrolase KIAA1363 that is diethyl phosphorylated in mouse brain by chlorpyrifos oxon. Chlorpyrifos is a unique OP because it is highly lipophilic, and its leaving group is also implicated in certain toxicities. Blood acylpeptide hydrolase activity has been identified as a possible biomarker of OP exposure (Murray et al. 2005; Richards et al. 1999). In a number of detailed studies, Costa and colleagues advanced paraoxonase (PON1) and its isoforms as biomarkers of susceptibility to OP toxicity (Costa 1998; Costa et al. 2003, 2005). In most studies, one protein or pathway is identified to serve as a biomarker. But the measurement of one OP protein target, although valuable, can be questionable unless background exposure levels are relatively stable over time. Moreover, as outlined above, the OP-adduction step may not be the sole indicator of an interaction. This limitation holds true also for the traditional cholinesterase tests, metabolite analysis, and direct measurements.

Molecular probes such as FP-biotin (Figure 9) may serve as useful tools in analyzing multiple proteins in complex protein samples (Adam et al. 2001, 2002; Grigoryan et al. 2008; Kidd et al. 2001; Liu et al. 1999). The probe design contains a reactive OP moiety containing a fluoro leaving group and a biotin “catch” group connected by a long linker group to allow the reactive OP to reach deep into protein structures. Ideally, a proteomic experiment will provide protein identification, expression levels, and the functional state of proteins. In studies aimed at profiling serine hydrolase activity, FP-biotin probes were used to selectively isolate and identify serine hydrolases in crude cell and tissue extracts and also for the functional characterization of these enzymes (Kidd et al. 2001; Liu et al. 1999) (Figure 9). Although FP-biotins are excellent probes of serine hydrolases and other OP-reactive proteins, the long lipophilic tethers and attenuated reactivity as a phosphonate compared with phosphate likely select for a protein population different from that of the OP oxon structures. Thus, differences in reactivity and cell permeability properties of FP-biotin probes must be considered when assessing possible OP–protein biomarkers.

In one study, Kidd et al. (2001) investigated the rate differences in which the serine hydrolases react with FP-biotin probes after treatment with oleoyl trifluoromethyl ketone (OTFMK), a reversible inhibitor of serine hydrolases. Serine hydrolase targets were identified directly in complex proteomes by comparing the rates of binding of the serine hydrolases to the FP-biotin probes in the control and OTFMK-treated samples (Kidd et al. 2001). An *in vitro* assay exposing purified tubulin to an OP–biotin probe demonstrated not only that OPs bind this previously unidentified target, but also that they bind tyrosine residues in tubulin. The OP-reactive tyrosine residues reside either near the GTP binding site or within loops that interact laterally with protofilaments, indicating that this binding, if it occurs *in vivo*, may lead to noncholinergic toxic outcomes (Grigoryan et al. 2008). Lockridge and colleagues (Ding et al. 2008; Schopfer et al. 2005a, 2005b) also used FP-biotin combined with MS to identify OP-modified proteins in human plasma. The plasma was treated with FP-biotin; the proteins were separated into low- and high-abundance portions and digested with trypsin; and the FP-biotinylated peptides were isolated by binding to avidin beads. Proteins identified in these studies included albumin, ES1 carboxylesterase, propionyl and methylcrotonyl coenzyme A carboxylase-α, and pyruvate carboxylase. The investigators were able to readily identify FP-biotin–labeled albumin because of its high concentration in human plasma (Ding et al. 2008; Schopfer et al. 2005a, 2005b). The results demonstrated that albumin is an OP scavenger and undergoes a covalent reaction with OP on five tyrosines and two serines (Aardema and MacGregor 2002; Peeples et al. 2005; Schopfer et al. 2005a, 2005b; Wieseler et al. 2006). Although the FP-biotin probe was unable to identify any additional novel OP protein targets, the results from this study suggest that OP–albumin adducts could be used to monitor OP exposure (Ding et al. 2008).

FP-biotin has a relatively large structure compared with OPs, so it can be argued that this probe may react differently with various proteins in biological systems and possibly fail to detect important OP protein targets. This could prove to be problematic with enzymes such as AChE and BChE that contain OP binding sites located deep within the molecule. Schopfer et al. (2005a, 2005b) investigated the rates of reactivity and binding affinity of FP-biotin with AChE and BChE. The results from their study demonstrate that, despite its large biotin group, FP-biotin reacts with both AChE and BChE at rates comparable to reaction rates of other OPs. Therefore, the authors concluded that FP-biotin is a potent OP and can serve as a useful probe for identifying OP protein targets. Taken together, the studies described here demonstrate that molecular probes such as biotinylated OPs can be powerful tools for proteomic studies. Although FP-biotin does not precisely simulate OP structure, the ability to rapidly isolate and identify labeled proteins in crude cell and tissue extracts, as well as provide insight into posttranslational events that regulate protein function, outweighs some of the potential limitations.

## Conclusions

The mechanisms by which OP insecticide oxons covalently adduct AChE, BChE, and other serine hydrolases have been relatively well understood, but many of the postinhibition processes have not been well characterized. Advances in protein MS have clearly aided investigators with a new and powerful tool to interpret not only the adduction event but also the molecular steps that occur after adduction. As a result, it is now somewhat routine to investigate not only OP–ChE adduct formation but also each step in the process that leads to injurious modifications of the protein. With these important steps accomplished, investigators can now broaden the scope of their inquiry into how proteins other than cholinesterases are modified by OP compounds and, hence, how the connected cellular level events may be compromised.
